# Active Use of Parks in Flanders (Belgium): An Exploratory Observational Study

**DOI:** 10.3390/ijerph14010035

**Published:** 2016-12-30

**Authors:** Linde Van Hecke, Jelle Van Cauwenberg, Peter Clarys, Delfien Van Dyck, Jenny Veitch, Benedicte Deforche

**Affiliations:** 1 Department of Public Health, Faculty of Medicine and Health Sciences, Ghent University, 9000 Ghent, Belgium; Jelle.VanCauwenberg@ugent.be (J.V.C.); Benedicte.Deforche@ugent.be (B.D.); 2 Physical Activity, Nutrition and Health Research Unit, Department of Movement and Sport Sciences, Faculty of Physical Education and Physical Therapy, Vrije Universiteit Brussel, 1050 Brussels, Belgium; peter.clarys@vub.ac.be; 3 Department of Movement and Sport Sciences, Faculty of Medicine and Health Sciences, Ghent University, 9000 Ghent, Belgium; Delfien.VanDyck@UGent.be; 4 Fund for Scientific Research Flanders (FWO), 1000 Brussels, Belgium; 5 Institute for Physical Activity and Nutrition (IPAN), School of Exercise and Nutrition Sciences, Deakin University, Geelong Waurn Ponds Campus, Geelong, VIC 3216, Australia; jenny.veitch@deakin.edu.au

**Keywords:** direct observation, recreation, physical activity, SOPARC, active living

## Abstract

Parks have the potential to increase physical activity at the community level by providing opportunities to be active. In order to inform interventions to promote physical activity in parks, insight is needed concerning park user characteristics, the activity level of park users, the types of activities performed and associations between park areas and temporal variables with observed physical activity levels. Park user characteristics (sex, age, ethnicity and activity level) were recorded within pre-defined park areas in two parks in Ghent (Belgium) using the System for Observing Play and Recreation in Communities (SOPARC). Most park users were male, adult, and engaged in vigorous-intensity physical activity (48%). Most popular activities were biking (38%), sitting (23%) and walking (15%); accordingly, trails were used most and had the highest levels of physical activity compared to other park areas. Parks were used least frequently in the morning, during the weekend and by seniors. Therefore, active park use during morning periods, on weekend days and by seniors should be promoted and urban planners should consider that different park areas can possibly elicit varying activity levels among park users.

## 1. Introduction

Worldwide, the number of people with chronic diseases and who are overweight or obese increases continuously [1,2]. Previous research has shown that regular physical activity can prevent becoming overweight or obese [3,4] and is related to a lower incidence of chronic diseases [5]. However, most people do not meet physical activity recommendations [6,7]. Therefore, there is an urgent need for interventions promoting physical activity to enhance population health [5,8]. Previous research suggests that parks can play an important role in promoting physical activity among people of all ages as parks are present in most communities and are generally free to use [9,10,11]. Parks can provide a suitable setting for organized and non-organized physical activity by providing a variety of physical activity facilities. In an urban environment, parks can also be a destination to visit by foot or bike [12,13] and thereby have the potential to increase physical activity even if park users engage in sedentary behaviour after arriving at the park.

A substantial amount of research has demonstrated that people living closer to parks perform more leisure-time and park-based physical activity [11,14,15,16,17,18]. Moreover, studies from Australia and the UK have shown that park users are more likely to comply to physical activity recommendations [19,20]. However, there seems to be a difference in physical activity levels depending on the kind of park area or facilities used (i.e., wooded area, tennis court, trails and paths, meadows, open spaces) [21,22,23,24]. For example, in the U.S., Besenyi and colleagues (2008) reported higher adult energy expenditure on paved trails and tennis courts than on open spaces, playgrounds and picnic areas. Among children, higher energy expenditure was observed in playgrounds compared to picnic shelters [21]. 

Moreover, Bedimo-Rung and colleagues emphasized in their conceptual model, that not only structural characteristics of parks, but also individual user characteristics influence park visitation and park-based physical activity (e.g., women are less likely to visit a park compared to men) [9]. Therefore, it would be useful to determine physical activity levels according to the specific park area used and to determine if differences exist based on gender and age of the park users. Park use and park-based physical activity can also differ between workweek and weekend days and according to the hour of the day (e.g., U.S. parks were used less frequently during the morning [11]). However, knowledge is limited in regards to the temporal aspects of park use. Therefore, insight is needed into the time of the day and day of the week during which parks are used less often so that they can be targeted in interventions aimed at increasing park use.

Objective information on park users can be obtained through direct observation tools, of which the System for Observing Play and Recreation in Communities (SOPARC) is the most frequently used [25,26]. Two recent systematic reviews of studies using SOPARC and other observational methods to measure park-based physical activity [26,27] indicated that most research originated from North America and few studies were conducted in Australia [28,29], South America (Brazil) [30,31], Asia (Taiwan, China) [32,33] and Europe (Denmark and Belgium) [34,35,36]. One study used direct observation to determine the association between park-based physical activity levels and neighbourhood walkability and income in Belgian and U.S. parks. However, they did not report the specific areas of the parks related to physical activity nor the time of the day and day of the week when parks were used most often [34]. Research on park visitation and park-based physical activity in Europe and Belgium is scarce and urban environments in Europe differ from those in other parts of the world. Accordingly, insight is needed on this topic in order to better understand park user characteristics, to define priorities for park renewal and construction, and to inform interventions to promote physical activity in parks.

Therefore, the first aim of this study was to describe the characteristics of park users in Ghent (Flanders, Belgium), the activity levels of park users and the types of activities performed. Secondly, this study aimed to examine the association between park areas, day and time of day, and the observed number of park users and physical activity levels of park users for males and females and children/adolescents and adults/seniors using direct observation methods.

## 2. Materials and Methods 

### 2.1. Study Setting

Data collection occurred in two parks in Ghent (Flanders, Belgium) from July to October 2014. The city of Ghent has an area of 157.96 km² and has 253,266 inhabitants (population density: 1603 inh/km²) [37], of which 11.6% are under 9 years, 9.7% are 10−19 years, 56.9% are 20−59 years and 21.7% are older than 60 years [38]. The Ghent population consists of 49.4% males [38] with 18.8% part of an ethnic-cultural minority, mostly of Turkish or Bulgarian origin [39]. This study was approved by the Ethical Committee of the University Hospital in Ghent (2015/0550). The two parks comprised areas of 51,313 m² and 31,502 m² respectively, and included a variety of features/amenities (i.e., wooded area (=area with lots of trees), grassy area with a playground, pond, trails, etc.). 

### 2.2. Measurements

#### 2.2.1. The Environmental Assessment of Public Recreation Spaces (EAPRS)

The Environmental Assessment of Public Recreation Spaces (EAPRS) tool was used to provide an overview of the park amenities and features. This tool is a comprehensive audit instrument that characterizes the physical environment within public parks [40]. This audit was completed by a researcher prior to the observations. 

#### 2.2.2. The System for Observing Play and Recreation in Communities (SOPARC)

The System for Observing Play and Recreation in Communities (SOPARC) was used to obtain direct information on park use and characteristics of the park users. SOPARC has been proven to be a valid and reliable observation tool [25]. Before the observations were conducted, both parks were divided into observable target areas (5−7 areas per park) which represented specific locations within the parks (e.g., wooded area, grassy area with a playground, ponds, trails). On-site observations were conducted for nine days (simultaneous measurements at the two parks), including five weekdays and four weekend days, on different days of the week and at varying times of the day (7:30, 12:30, 15:30, 18:30) [41]. Each observation moment lasted approximately 20−30 min. At these predetermined time points, trained researchers scanned each target area and recorded the following characteristics of the park users: age (child = 0−12 years, adolescent = 13−20 years, adult = 21−59 years and senior ≥60 years), gender, ethnicity (Caucasian or non-Caucasian) and activity level (sedentary, moderate or vigorous). Additionally, each area was assessed on its accessibility, usability (e.g., not excessively wet or roped off for repair), equipment (e.g., balls, jump ropes), supervision (adults that are either payed, or volunteer to supervise in a park), provision of lighting and organization of activities. In total, 432 scans were completed across 12 target areas (5−7 areas per park) during 36 observation moments (nine days × four time points) in each park. Observations were only conducted in neutral to good weather (i.e., no rain) and cancelled observations due to bad weather were rescheduled in the following week on the same day of the week. Three researchers (Linde Van Hecke, Lars Van Elewijck, Silke Van Hoof) conducted the observations; however, only a single observer was present in each park on each day. One researcher (Linde Van Hecke) conducted the observations on all nine days in one of the two parks. The other two researchers (Lars Van Elewijck and Silke Van Hoof), each performed observations on four and five days, respectively. 

All target areas were accessible and usable but none were supervised or equipped and all observations took place during daylight hours. Therefore, these variables were not included in the analysis. The variable “organized activities” was also not included in the analyses because of the low prevalence of observing organized activities in the parks (2.3% of observation moments). The target areas which included ponds also had low usage (1.4% of all observation moments) so were not included in the analyses.

#### 2.2.3. Training of the Researchers

The researchers were trained using the SOPARC protocol and training video [42]. The researchers conducted ten test observations in each park (20 in total) which consisted of independent observations that were carried out simultaneously. These were used to establish interrater reliability (IRR) on park user characteristics (i.e., gender, age, ethnicity and level of physical activity) and park characteristics (i.e., accessibility, usability, equipment, supervision, lighting and organized activities). Reliability was defined as good for ICCs ranging from 0.60 to 0.74 and excellent for ICCs higher than 0.75 [43]. Agreement between observers was good to excellent for all user characteristics (ICC = 0.74−1.00). Agreement for the park characteristics was also good to excellent, with the lowest ICC for usability of the target area (ICC = 0.65). As recommended by Hallgren (2012), all IRR estimates from the test observations had to be good or excellent (>0.60) before the actual observations commenced [43].

### 2.3. Analyses

In order to analyse the mean physical activity level in each target area during an observation moment, Metabolic Equivalents (METs)/observation/target area were computed for all users, and for the different age (children and adolescents, adults and seniors) and gender subgroups within this area. To do so, the total observed number of park users in each activity level (i.e., sedentary, moderate or vigorous), was multiplied with the corresponding MET value (sedentary = 1 MET, e.g., sitting reading a book; moderate = 3 METs, e.g., walking; vigorous = 6 METs, e.g., jogging). The result of all activity levels was summed and then divided by the total number of observed park users in this area to obtain the mean activity level per person. This approach has previously been used by Van Dyck et al. 2013 [34]. 

In order to gain insight in gender and age-specific differences in the number of park users and physical activity levels according to the park area, hour of the day and day of the week, analyses were performed for males and females, and due to limited power, for youth (0−20 years) and adults (>20 years) separately rather than for the four age groups. Since all variables were measured at the park area level, it was not possible to examine interaction effects. 

Associations of the independent variables (park area and temporal characteristics: week or weekend day, time of the day) with the dependent variables (number of park users and physical activity levels) were examined using multilevel Hurdle models (level 1 = h, level 2 = day, level 3 = target area, level 4 = park) using the package LME4 [44] in R version 3.1.0 (RStudio Team (2016). RStudio: Integrated Development for R. RStudio, Inc., Boston, MA, USA). Hurdle models were used because of the high number of zero observations (i.e., empty target areas). Hurdle models consist of two parts. First, the association between the independent variables and observing at least one park user in the park area was estimated by means of logistic regression analysis (binomial variance and logit link function). This regression coefficient represents the association between the independent variables and the odds of observing a target area where at least one park user was present. Secondly, the model estimated the association between the independent variables and the number of users/activity level for the park areas that were not empty. This regression coefficient represents the proportional change in number of users/activity level of the park users associated with a one-unit difference in the independent variables for park target areas where at least one person was present. Based on the Akaike’s Information Criteria (AIC), the variance and link function that best fitted the data were defined (Poisson variance with log link function for count variables, and Gamma variance with log link function for continuous dependent variables). No logistic regression analyses were performed for the dependent variable “activity level”, as these would yield identical results as the analysis on the presence of at least one park user. Level of significance was set at α = 0.05. 

## 3. Results

### 3.1. Descriptive Statistics

In total, 837 park users were observed of which the majority were adults (43.9%), 20.7% were children (0−12 years), 25.8% were adolescents (13−20 years) and only 9.7% were aged over 60 years. The majority of participants were male (58.7%), except for the adult category, where gender was distributed equally. Users were predominantly Caucasian (82.8%), almost half were vigorously active (48.2%), 24.7% were moderately active and 27.1% were sedentary (Table 1). Park characteristics assessed using the EAPRS tool are presented in Table 2. The activities that were observed include biking (37.9%), sitting (22.5%), walking (15.1%), playing (7.3%), walking the dog (5.4%), ball sports (5.0%), jogging (3.4%), standing (3.2%), and lying down (0.4%). However, during 57.6% of the observation scans, the target areas had no park visitors present.

### 3.2. Associations of the Park Areas and Temporal Characteristics with the Odds of Observing at Least One Park User

The logistic regression model (Table 3) for the total sample shows that the odds of observing at least one park user were higher at midday, in the afternoon and in the evening compared to morning observations. Separate analysis revealed similar results for males and females (not at midday) and for children/adolescents and adults/seniors. The odds of observing at least one park user were higher on trails compared to grassy areas with a playground. This was found in all separate subgroups. The odds of observing at least one park user were lower for wooded areas compared to a grassy area with a playground. When groups were analysed separately, similar results were found for all groups. Compared to a weekday, the odds of observing at least one park user on a weekend were lower. This was only found for adults/seniors in the separate analyses but not for children/adolescents nor when males and females were analysed separately.

### 3.3. Association of the Park Areas and Temporal Characteristics with the Number of Park Users

This section addresses the results of the second part of the Hurdle models (Poisson models in Table 3) and represents the proportional change in the number of park users associated with the independent variables for park target areas where at least one person was present. 

In the total sample, no differences were found in the number of park users during midday compared to morning observations, nor for males, children/adolescents and adults/seniors. However, 57% more females were observed at midday compared to morning observations (95% CI = 1.1−2.3). In the total sample, 109% more park users were observed in the afternoon compared to morning observations (95% CI = 1.7−2.7). This was also found for males, females and children/adolescents but not for adults/seniors. In the total sample, 40% more park users were observed in the evening compared to morning observations (95% CI = 1.1−1.8). This was also found for children/adolescents but not for adults/seniors, females or males. On trails, compared to a grassy area with a playground, no differences were found in the number of park users for the total sample, females and children/adolescents. However, more males and adults/seniors were observed on trails than grassy areas with a playground. In wooded areas, there were 55% (95% CI = 0.3−0.7) less park users compared with grassy areas with a playground. Similar results were found in male and adults/seniors subgroups, but these differences were not observed among females and children/adolescents. No significant differences were found in the number of park users during the weekend compared to weekdays.

### 3.4. Association of the Park Areas and Temporal Characteristics with Park Users’ Activity Levels

The following section describes the results of the Gamma models (Table 4). These analyses were performed for park areas where at least one person was present. In the total sample, among males, children/adolescents and adults/seniors, no differences were found in observed activity levels between morning and midday. The activity level among females was 24% (95% CI = 0.6−1.0) lower at midday compared to the morning. The activity level of the total sample was 24% (95% CI = 0.6–0.9) lower in the afternoon compared to morning observations. Similar results were found for females and adults/seniors, but not for males and children/adolescents. No difference in activity level was found between evening and morning observations. Activity levels for the total sample (and all subsamples) was 73% (95% CI = 1.5–2.1) higher on trails compared to grassy areas with a playground. No difference in activity level was found between wooded areas compared to grassy areas with a playground for the total sample and all subsamples. Analyses of the total sample, females, children/adolescents and adults/seniors revealed no difference in activity level between the weekend and weekdays. Activity level among males was 21% higher in the weekend (95% CI = 1.0−1.4).

## 4. Discussion

Sufficient physical activity contributes to better health status across the lifespan, and parks have the potential to increase physical activity at the community level by providing opportunities for physical activity. This study described the characteristics of park users in Ghent (Flanders, Belgium), the activity levels of park users, the type of activities performed and the associations between park areas and temporal variables with observed physical activity levels. The majority of the observed visitors were males, adults and were engaged in vigorous-intensity physical activity with cycling, sitting and walking being the most popular activities. Seniors were the least represented age group and the smallest number of park visitors were observed in the morning and during weekends. Activity levels were higher on trails and lower in wooded areas compared to grassy areas with a playground. 

Overall, the parks in this study had low levels of visitation (58% of observation moments, parks were empty), which could possibly be caused by the small amount of features and amenities present in the parks (according to the audit that was performed). However, these parks were selected because they did not have atypical features (for Flanders) and had not been renovated recently. Moreover, organized activities were limited and supervision and provision of equipment was lacking. This indicates that there are many opportunities to improve the attractiveness, use and physical activity within these parks. When developing programs to increase park visitation, it is important to determine which specific target groups are currently underrepresented and could benefit from extra attention, and which park areas are associated with higher activity levels. In addition, associations of park areas and temporal variables with the number of park users and physical activity levels were examined according to gender and age group.

Most park users in this study were male (58.7%), which is consistent with previous research using observational measurements in Belgium [34] and with the results of a review by Evenson and colleagues [27]. However, this gender difference was only present for children, adolescents and seniors, and not for adults, where the distribution was more equal between males and females. For children and adolescents, this gender difference may be attributed to higher independent mobility among boys compared to girls [45] or by the nature of the activities boys and girls participate in (i.e., girls like to go shopping, whereas boys prefer to do sports [46]). The age distribution of the park users was comparable with those from observational research from the U.S., with the majority of park users being adults and only a minority being seniors. In this study, more adolescents were observed than in U.S. parks [11,25,34,47,48] and Australian parks [28], but less adolescents than in previous Belgian research [34]. The relatively low percentage of seniors observed in the parks (9.7% of the park users were older than 60, compared to 21.7% of the Ghent population [38]) indicates that extra effort may be needed to encourage seniors to use parks more often. The city of Ghent has many parks spread throughout the city and they should therefore be readily accessible or within close proximity to home for most residents. However, the current park features/amenities may not encourage seniors to visit the parks or access to the parks may be difficult (i.e., uneven footpaths [49]) and discourage visitation. Future studies need to explore which park features are important for park use among seniors. Of the park users observed, 82% were Caucasian, which is representative of the Ghent population and similar to previous research [34,39]. 

In this study, almost half of the observed park users were observed engaging in vigorous-intensity physical activity, whereas in previous observational studies in the U.S., most park users were sedentary [11,21,22,25,34,47,48]. In Australia, most park users were standing or moderately active [28]. Sedentary activities in parks can have social benefits [50] and even people who use parks for sedentary activities may travel to the park using active transport such as by foot or bike [12,13]. Previous research in adolescents indicated that adolescents prefer to alternate between active and sedentary activities [51]. Therefore, parks should be designed so that they support both physical activity and sedentary activities and provide sufficient infrastructure to support active travel to the park. The high number of vigorously active users in this study could possibly be attributed to the high number of cyclists observed in the parks (38% of park users were cycling). However, it must be noted that most cyclists were only passing through the park to travel elsewhere suggesting that the parks offered an alternative, convenient and/or safe cycling route. Policy makers should take this into account, as cycling is often forbidden in parks. At least one cycle lane in each park could be a useful strategy for future park development. The other activities that were frequently observed in the parks in this study were sitting (23% of users), walking (15% of users) and playing active games such as hide and seek (7% of users). This could indicate that apart from using a park to cycle through, parks in Flanders are used mostly as a place to relax rather than to engage in intensive sports or to work out, which is consistent with the conclusion by Van Dyck et al. (2013) [34]. However, the qualitative results of a study conducted by Cohen and colleagues (2007) revealed that U.S. parks were used for sedentary activities such as picnics (22% of users), as well as for more active purposes such as playing basketball (15% of users) or as spectators of an organized activity (13% of users) [11]. This indicates that cultural differences may exist in park activities between Europe and the U.S. 

Less participants were observed during morning periods and during weekends. However, the activity levels were lower during the day (for females and all age groups) and higher on the weekend for males. This may indicate that when less people are present in the park, park users are more likely to engage in physical activities instead of sitting behaviours, or park users may have different intentions when they visit a park in the morning (e.g., they are cycling to school/work) compared to during the day or during the weekend compared to a weekday. Other studies have also shown park visitation to be lower in the morning [11,28]. However, an Australian study on park use in metropolitan parks reported more visitors during the weekends [28] than on weekdays, which is contradictory to our results. These contradicting results could be due to differences in size and location of the parks included in the Australian study: large metropolitan parks outside of the city (=long travel time) compared to small parks located in urban areas. If the findings of the current study are confirmed in future research for small parks, future interventions promoting park use in small urban parks could possibly target morning and weekends as these are the moments small urban parks are currently used least. 

The odds of at least one park user being present were higher on trails and lower in wooded areas compared to grassy areas with a playground. No difference was found in the number of children/adolescents observed on trails and in wooded areas compared to grassy areas with a playground. This is surprising as it could be hypothesized that children and adolescents would use a grassy area with a playground more often than a trail [21,52]. This may be the result of analysing children and adolescents together, thereby age-dependent differences may have been overlooked. Future research should allow for analyses for each age group separately by including more parks. Furthermore, the activity level of all user groups was higher on trails compared to grassy areas with a playground. This could possibly be due to the high number of cyclists on the trails. This supports previous research from Canada and Denmark that revealed a significant association between the presence of a walking/cycling path and park-based physical activity in adults [53,54] and research from Besenyi et al. (2013) that revealed the highest energy expenditure among adults on paved trails in U.S. parks [21]. A possible approach for future urban planning may be to link existing urban parks with each other using trails to encourage walking and cycling [53,55]. This could be a suitable strategy for European historical cities such as Ghent, which are often densely built with many individual small urban parks. 

The higher activity level of children and adolescents on trails compared to grassy areas with a playground could be explained by the nature of the playgrounds. In the studied parks, the playgrounds consisted of components that primarily facilitate sedentary behaviour (such as a sand pit) rather than encourage physical activity. Furthermore, playgrounds are often designed for younger children [51]. Hence, playgrounds that include equipment such as climbing structures or basketball hoops that encourage physical activity should be chosen over playgrounds that include equipment that promotes sedentary behaviour [56]. Previous qualitative research in adolescents indicated that adolescents often visit public open spaces with younger siblings [51]. Therefore, it is also important that playgrounds are designed to cater for the needs of multiple age groups. 

A possible explanation for higher activity levels of females on trails compared to grassy areas with a playground could be that when females are present at a grassy area with a playground, they often engage in standing and sedentary activities to supervise children at the playground [11]. Cohen and colleagues (2007) have recommended the provision of equipment that provides opportunities for carers of children to be active near playgrounds while supervising their children (e.g., walking paths around the playgrounds, or adult fitness stations) [11]. This could be a valuable strategy to increase physical activity levels in parks among carers of children. 

Earlier findings highlight the importance of the presence of trees in parks as this is associated with physical activity [54,57], mental health [58] and the provision of shade [59]. However, in this study, the wooded areas had the lowest number of users (55% lower than trails). This low number of users may be due to the density of the wooded area (in this study the wooded area had a high density of trees) which may have a negative influence on park users’ perceptions of safety, since secluded areas may create feelings of insecurity [57,60]. Therefore, park designers should ensure the presence of trees but avoid creating densely wooded areas. 

### 4.1. Strengths

This study used a valid and reliable observation tool (designed specifically for parks) to assess park use, park-based physical activity and characteristics of the park users [25]. All observers received training to use this tool and interrater reliability was confirmed before starting the observations. Additionally, the same observers performed the observations in both parks at exactly the same time and on the same days. Cohen et al. (2011) recommended at least four days of observation to obtain robust measures of park use and user characteristics [41]. In this study, observations were performed on nine days. 

### 4.2. Limitations

This study only comprised two parks in Ghent and the findings should be considered exploratory. The audit of park characteristics was not validated by a second observer, and this should be considered as a limitation of the study. In the future, park audits should include an assessment of the park areas separately and an assessment of the quality and condition of the park features and amenities. In addition, to provide a better understanding of the results, future research could include qualitative methods to study the perceptions of park visitors. The observations only took place during neutral to good weather, therefore no conclusions could be drawn about the association between weather and park use. In addition, the observations were performed in summer and beginning of autumn (July to October) so no seasonal effect could be examined and results may be different in other seasons. The observations took place at fixed moments in time on nine days and other days and moments could possibly provide different results. Further, no additional information (such as living close to the park, income or transport mode to the park) was collected from the park users. A final methodological limitation is the estimation of the park users’ age which may have led to the misclassification of park users into different age groups. 

## 5. Conclusions 

Flemish parks provide many opportunities for physical activity at the community level but are currently underutilized. More specifically, (active) park use during morning periods and weekends and by all age and gender groups with special attention to seniors should be promoted. Furthermore, trails were found to be the park area that was used most and at the highest level of physical activity. When designing or renewing urban parks, urban planners and policy makers should take into account that different park areas may encourage varying physical activity levels among park users and playgrounds may need to be designed or refurbished in order to encourage physical activity. Future research should build on this exploratory research by including a larger number of parks with greater variety of park areas and by conducting natural experiments to gain insight into the causal relationship between specific park characteristics, park visitation and park-based physical activity.

## Figures and Tables

**Table 1:** Observed park user characteristics.

Park User Characteristics	Total		Children		Adolescents		Adults		Seniors	
	n	%	n	%	n	%	n	%	n	%
Park users	837		173	20.7	216	25.8	367	43.9	81	9.7
Male	491	58.7	117	67.6	140	64.8	187	51.0	47	58.0
Female	346	41.3	56	32.4	76	35.2	180	49.1	34	42.0
Caucasian	693	82.8	145	83.8	173	80.1	303	82.6	72	88.9
Non Caucasian	144	17.2	28	16.2	43	19.9	64	17.4	9	11.1
Sedentary	227	27.1	35	20.2	71	32.9	101	27.5	20	24.7
Moderately active	207	24.7	55	31.8	49	22.7	75	20.4	28	34.6
Vigorously active	403	48.2	83	48.0	96	44.4	191	52.0	33	40.7

**Table 2:** Descriptive park characteristics using the EAPRS audit tool.

Descriptive Characteristics	Park 1	Park 2
Access		
Access to the park is free	Yes	Yes
Neighbourhood immediately surrounding park	Residential	Residential
Entrances	3	3
Bike racks	No	Yes
Parking lots	Yes	No
Sidewalks adjacent to park	Yes	Yes
Roadways trough park	No	No
Trails & paths		
Paved trails present	No	Yes
Unpaved trails	No	Yes
Paths	Yes	Yes
General areas		
Open space	Yes	Yes
Meadows	No	No
Wooded area	Yes	Yes
Pond	Yes	Yes
Water Areas		
Stream/creek	No	No
Swimming pool	No	No
Fountain	No	No
Beach area	No	No
Eating/drinking features		
Water fountain	No	No
Grill/fire pit	No	No
Picnic area	No	No
Vending food/drinks	No	No
Facilities		
Restrooms	No	No
Shelter	No	No
Entertainment venues	No	No
Historical features	No	No
Sitting and resting features (non-trail)		
Benches	Yes	No
Tables	No	Yes
Seat walls	No	No
Bleachers	No	No
Landscaping & General Aesthetics		
Flowers	No	No
Shrubs/bushes	No	No
Landscaping beds	No	No
Views of outside park	No	No
Sculpture of other art	No	No
Trash cans	Yes	Yes
Wildlife area	No	No
Information related features		
Rules/regulation signs	No	No
Maps	Yes	No
Event postings	Yes	Yes
Safety related features		
Telephone	No	No
Play structure & other play components		
Play structure present	Yes (1)	Yes (2)
Seating around play structure	Yes	Yes
Separate play sets for different age groups	No	No
Surface material	Sand	Sand
Things to hang from	Yes	No
Things to slide down	Yes (1)	Yes (1)
Things to climb on, up, or through	Yes (2)	Yes (1)
Things to stand or walk on	No	No
Things to spin	Yes (1)	No
Swings	Yes (1 baby, 1 chair)	Yes (2 chair)
Spring toys	Yes (3 animals)	Yes (2 animals)
Imaginary play structure	No	No

**Table 3:** Hurdle models.

Independent Variables	Total Number of Park Users				Male Park Users				Female Park Users			
	Logistic Regression ^a^		Poisson Model ^b^		Logistic Regression ^a^		Poisson Model ^b^		Logistic Regression ^a^		Poisson Model ^b^	
	OR	95% CI	Exp. B	95% CI	OR	95% CI	Exp. B	95% CI	OR	95% CI	Exp. B	95% CI
Time of day (ref = morning)												
lunch	4.40 ***	1.94–9.99	0.98	0.76–1.29	7.29 ***	3.01–17.68	0.71	0.49–1.02	1.38	0.63–3.03	1.57 *	1.06–2.33
afternoon	6.82 ***	2.96–15.62	2.09 ***	1.65–2.66	15.46 ***	6.21–38.48	1.53 *	1.10–2.12	3.76 ***	1.72–8.23	2.01 ***	1.40–2.88
evening	4.46 ***	1.97–10.09	1.40 **	1.09–1.81	6.01 ***	2.50–14.42	1.14	0.81–1.61	3.26 **	1.49–7.10	1.36	0.92–1.99
Type of park area (ref = grassy area with a playground)												
trail	13.09 ***	4.24–40.41	1.79	0.90–3.59	15.79 ***	4.38–56.96	2.09 *	1.12–3.92	4.53 *	1.37–14.96	1.43	0.96–2.11
wood	0.05 ***	0.02–0.14	0.45 ***	0.31–0.67	0.06 ***	0.02–0.15	0.65 *	0.43–0.97	0.02 ***	0.00–0.12	0.55	0.18–1.69
Day type (ref = week)												
weekend	0.38 *	0.15–0.98	0.96	0.63–1.45	0.72	0.26–1.92	1.09	0.72–1.66	0.46	0.20–1.06	0.75	0.53–1.07
**Independent Variables**	**Child/Adolescent Park Users**				**Adult/Senior Park Users**							
	**Logistic Regression ^a^**		**Poisson Model ^b^**		**Logistic Regression ^a^**		**Poisson Model ^b^**					
	**OR**	**95% CI**	**Exp. B**	**95% CI**	**OR**	**95% CI**	**Exp. B**	**95% CI**				
Time of day (ref = morning)												
lunch	4.86 **	1.71–13.79	2.05	0.96–4.34	3.05 **	1.35–6.88	0.89	0.66–1.21				
afternoon	17.81 ***	6.35–49.98	4.91 ***	2.38–10.11	4.54 ***	2.00–10.29	1.17	0.88–1.54				
evening	10.78 ***	3.86–30.06	3.28 **	1.56–6.89	2.45 *	1.09–5.50	1.09	0.82–1.46				
Type of park area (ref = grassy area with a playground)												
trail	4.26 ***	1.81–10.05	1.66	0.82–3.34	8.15 ***	2.65–25.00	1.51 *	1.08–2.11				
wood	0.09 ***	0.03–0.23	0.67	0.43–1.05	0.04 ***	0.01–0.12	0.53 *	0.31–0.92				
Day type (ref = week)												
weekend	1.37	0.49–3.83	0.82	0.48–1.40	0.29 **	0.13–0.68	0.96	0.77–1.20				

^a^ The logistic regression model estimated the association of the independent variables with the odds of observing park users in the park areas. ^b^ The Poisson models (Exp. B) estimated the proportional difference in number of users associated with a one-unit difference in the independent variables for park target areas where at least one person was present. OR = Odds Ratio, CI = Confidence interval, Exp. B = Exponent of B, ref = reference category. * α < 0.05, ** α < 0.01, *** α < 0.001.

**Table 4:** Gamma models.

Independent Variables	Average PA Level for All Park Users		Average PA Level for Male Park Users		Average PA Level for Female Park Users		Average PA Level for Child/Adolescent Park Users		Average PA Level for Adult/Senior Park Users	
	Exp. B	95% CI	Exp. B	95% CI	Exp. B	95% CI	Exp. B	95% CI	Exp. B	95% CI
Time of day (ref = morning)										
lunch	0.85	0.69–1.04	1.02	0.79–1.30	0.76 *	0.59–0.98	0.83	0.52–1.33	0.80	0.64–1.00
afternoon	0.76 **	0.62–0.93	0.87	0.69–1.11	0.78 *	0.61–0.98	0.69	0.45–1.07	0.76 *	0.62–0.95
evening	0.87	0.71–1.07	0.86	0.67–1.11	0.91	0.73–1.17	0.78	0.50–1.21	0.83	0.66–1.04
Type of park area (ref = grassy area with a playground)										
trail	1.73 ***	1.45–2.06	1.96 ***	1.67–2.30	1.69 ***	1.37–2.08	1.54 **	1.19–2.00	1.92 ***	1.58–2.34
wood	1.01	0.78–1.32	1.01	0.80–1.28	1.29	0.85–1.94	0.99	0.69–1.43	1.05	0.79–1.41
Day type (ref = week)										
weekend	1.11	0.93–1.33	1.21 *	1.03–1.42	0.99	0.78–1.25	1.20	0.98–1.48	1.02	0.78–1.32

The Gamma models (Exp. B) estimated the proportional difference in activity level associated with a one-unit difference in the independent variables for park target areas where at least one person was present. All Gamma models were fitted using the log link function. PA = physical activity, Exp. B = Exponent of B, CI = Confidence interval, ref = reference category. * α < 0.05, ** α < 0.01, *** α < 0.001.

## References

[1] Alwan A. (2010). Global Status Report on Noncommunicable Diseases 2010.

[2] Mendis S. (2014). Global Status Report on Noncommunicable Diseases 2014.

[3] Janssen I., Katzmarzyk P.T., Boyce W.F., Vereecken C., Mulvihill C., Roberts C., Currie C., Pickett W. (2005). Comparison of overweight and obesity prevalence in school-aged youth from 34 countries and their relationships with physical activity and dietary patterns. Obes. Rev..

[4] Kesaniemi Y.A., Danforth E., Jensen M.D., Kopelman P.G., Lefebvre P., Reeder B.A. (2001). Dose-response issues concerning physical activity and health: An evidence-based symposium. Med. Sci. Sports. Exerc..

[5] Bauman A.E. (2004). Updating the evidence that physical activity is good for health: An epidemiological review 2000–2003. J. Sci. Med. Sport.

[6] Marques A., Sarmento H., Martins J., Saboga Nunes L. (2015). Prevalence of physical activity in European adults—Compliance with the World Health Organization’s physical activity guidelines. Prev. Med..

[7] Sisson S.B., Katzmarzyk P.T. (2008). International prevalence of physical activity in youth and adults. Obes. Rev..

[8] Warburton D.E., Nicol C.W., Bredin S.S. (2006). Health benefits of physical activity: The evidence. CMAJ.

[9] Bedimo-Rung A.L., Mowen A.J., Cohen D.A. (2005). The significance of parks to physical activity and public health: A conceptual model. Am. J. Prev. Med..

[10] Kaczynski A. (2007). Environmental correlates of physical activity: A review of evidence about parks and recreation. Leis. Sci..

[11] Cohen D.A., McKenzie T.L., Sehgal A., Williamson S., Golinelli D., Lurie N. (2007). Contribution of public parks to physical activity. Am. J. Public Health.

[12] Veitch J., Carver A., Hume C., Crawford D., Timperio A., Ball K., Salmon J. (2014). Are independent mobility and territorial range associated with park visitation among youth?. Int. J. Behav. Nutr. Phys. Act..

[13] Menai M., Charreire H., Feuillet T., Salze P., Weber C., Enaux C., Andreeva V.A., Hercberg S., Nazare J.A., Perchoux C. (2015). Walking and cycling for commuting, leisure and errands: Relations with individual characteristics and leisure-time physical activity in a cross-sectional survey (the ACTI-Cites project). Int. J. Behav. Nutr. Phys. Act..

[14] Babey S.H., Brown E.R., Hastert T.A. (2005). Access to safe parks helps increase physical activity among teenagers. Policy Brief UCLA Cent. Health Policy Res..

[15] Kaczynski A., Potwarka L.R., Smale B.J.A., Havitz M.E. (2009). Association of parkland proximity with neighborhood and park-based physical activity: Variations by gender and age. Leis. Sci..

[16] Sugiyama T., Francis J., Middleton N.J., Owen N., Giles-Corti B. (2010). Associations between recreational walking and attractiveness, size, and proximity of neighborhood open spaces. Am. J. Public Health.

[17] Van Cauwenberg J., Cerin E., Timperio A., Salmon J., Deforche B., Veitch J. (2015). Park proximity, quality and recreational physical activity among mid-older aged adults: Moderating effects of individual factors and area of residence. Int. J. Behav. Nutr. Phys. Act..

[18] Baker E.A., Schootman M., Kelly C., Barnidge E. (2008). Do recreational resources contribute to physical activity?. J. Phys. Act. Health.

[19] Veitch J., Ball K., Crawford D., Abbott G., Salmon J. (2013). Is park visitation associated with leisure-time and transportation physical activity?. Prev. Med..

[20] Flowers E.P., Freeman P., Gladwell V.F. (2016). A cross-sectional study examining predictors of visit frequency to local green space and the impact this has on physical activity levels. BMC Public Health.

[21] Besenyi G.M., Kaczynski A.T., Wilhelm S.S.A., Vaughan K.B. (2013). Demographic variations in observed energy expenditure across park activity areas. Prev. Med..

[22] Floyd M.F., Spengler J.O., Maddock J.E., Gobster P.H., Suau L.J. (2008). Park-based physical activity in diverse communities of two U.S. cities. Am. J. Prev. Med..

[23] Reed J., Arant C.A., Wells P., Stevens C., Hagen S., Harring H. (2008). A descriptive exmnination of the most frequently used activty settings in 25 community parks using direct observation. J. Phys. Act. Health.

[24] Shores K.A., West S.T. (2008). The relationship between built park environments and physical activity in four park locations. J. Public Health Manag. Pract..

[25] McKenzie T.L., Cohen D.A., Sehgal A., Williamson S., Golinelli D. (2006). System for observing play and recreation in communities (SOPARC): Reliability and feasibility measures. J. Phys. Act. Health.

[26] Joseph R.P., Maddock J.E. (2016). Observational park-based physical activity studies: A systematic review of the literature. Prev. Med..

[27] Evenson K.R., Jones S.A., Holliday K.M., Cohen D.A., McKenzie T.L. (2016). Park characteristics, use, and physical activity: A review of studies using SOPARC (system for observing play and recreation in communities). Prev. Med..

[28] Veitch J., Carver A., Abbott G., Giles-Corti B., Timperio A., Salmon J. (2015). How active are people in metropolitan parks? An observational study of park visitation in Australia. BMC Public Health.

[29] Veitch J., Ball K., Crawford D., Abbott G.R., Salmon J. (2012). Park improvements and park activity: A natural experiment. Am. J. Prev. Med..

[30] Parra D.C., McKenzie T.L., Ribeiro I.C., Ferreira Hino A.A., Dreisinger M., Coniglio K., Munk M., Brownson R.C., Pratt M., Hoehner C.M. (2010). Assessing physical activity in public parks in Brazil using systematic observation. Am. J. Public Health.

[31] Hino A.A.F., Reis R.S., Ribeiro I.C., Parra D.C., Brownson R.C., Fermino R.C. (2010). Using observational methods to evaluate public open spaces and physical activity in Brazil. J. Phys. Act. Health.

[32] Pleson E., Nieuwendyk L.M., Lee K.K., Chaddah A., Nykiforuk C.I., Schopflocher D. (2014). Understanding older adults’ usage of community green spaces in Taipei, Taiwan. Int. J. Environ. Res. Public Health.

[33] Tu H., Liao X., Schuller K., Cook A., Fan S., Lan G., Lu Y., Yuan Z., Moore J.B., Maddock J.E. (2015). Insights from an observational assessment of park-based physical activity in Nanchang, China. Prev. Med. Rep..

[34] Van Dyck D., Sallis J.F., Cardon G., Deforche B., Adams M.A., Geremia C., De Bourdeaudhuij I. (2013). Associations of neighborhood characteristics with active park use: An observational study in two cities in the USA and Belgium. Int. J. Health Geogr..

[35] Peschardt K.K., Schipperijn J., Stigsdotter U. (2012). Use of small urban green spaces (SPUGS). Urban For. Urban Green..

[36] Lindberg M., Schipperijn J. (2015). Active use of urban park facilities—Expectations versus reality. Urban For. Urban Green..

[37] Statistics Belgium 2015. http://statbel.fgov.be/nl/modules/publications/statistiques/bevolking/Bevolking_nat_geslacht_opp_bevolkingsdichtheid.jsp.

[38] Gent in CIJFERS. http://gent.buurtmonitor.be/.

[39] Statistics Belgium 2016. http://statbel.fgov.be/nl/binaries/1801_nl%20gent_en_gentgebruikers_digitaal_tcm325-244558.pdf.

[40] Saelens B.E., Frank L.D., Auffrey C., Whitaker R.C., Burdette H.L., Colabianchi N. (2006). Measuring physical environments of parks and playgrounds: EAPRS instrument development and inter-rater reliability. J. Phys. Act. Health.

[41] Cohen D.A., Setodji C., Evenson K.R., Ward P., Lapham S., Hillier A., McKenzie T.L. (2011). How much observation is enough? Refining the administration of SOPARC. J. Phys. Act. Health.

[42] ALR Protocol SOPARC. http://activelivingresearch.org/soparc-system-observing-play-and-recreation-communities.

[43] Hallgren K.A. (2012). Computing inter-rater reliability for observational data: An overview and tutorial. Tutor Quant. Methods Psychol..

[44] Bates D.M.M., Bolker B., Walket S. R Package LME4. https://cran.r-project.org/web/packages/lme4/index.html.

[45] Carver A., Timperio A.F., Crawford D.A. (2012). Young and free? A study of independent mobility among urban and rural dwelling Australian children. J. Sci. Med. Sport.

[46] Duzenli T., Bayramoglu E., Ozbilen A. (2010). Needs and preferences of adolescents in open urban spaces. Sci. Res. Essays.

[47] Cohen D.A., Marsh T., Williamson S., Derose K.P., Martinez H., Setodji C., McKenzie T.L. (2010). Parks and physical activity: Why are some parks used more than others?. Prev. Med..

[48] Kaczynski A.T., Stanis S.A.W., Hastmann T.J., Besenyi G.M. (2011). Variations in observed park physical activity intensity level by gender, race, and age: Individual and joint effects. J. Phys. Act. Health.

[49] Van Cauwenberg J., De Bourdeaudhuij I., Clarys P., Nasar J., Salmon J., Goubert L., Deforche B. (2016). Street characteristics preferred for transportation walking among older adults: A choice-based conjoint analysis with manipulated photographs. Int. J. Behav. Nutr. Phys. Act..

[50] Kaźmierczak A. (2013). The contribution of local parks to neighbourhood social ties. Landsc. Urban Plan..

[51] Van Hecke L., Deforche B., Van Dyck D., De Bourdeaudhuij I., Veitch J., Van Cauwenberg J. (2016). Social and physical environmental factors influencing adolescents’ physical activity in urban public open spaces: A qualitative study using walk-along interviews. PLoS ONE.

[52] Floyd M.F., Bocarro J.N., Smith W.R., Baran P.K., Moore R.C., Cosco N.G., Edwards M.B., Suau L.J., Fang K. (2011). Park-based physical activity among children and adolescents. Am. J. Prev. Med..

[53] Kaczynski A.T., Potwarka L.R., Saelens B.E. (2008). Association of park size, distance, and features with physical activity in neighborhood parks. Am. J. Public Health.

[54] Schipperijn J., Bentsen P., Troelsen J., Toftager M., Stigsdotter U.K. (2013). Associations between physical activity and characteristics of urban green space. Urban For. Urban Green..

[55] Giles-Corti B., Broomhall M.H., Knuiman M., Collins C., Douglas K., Ng K., Lange A., Donovan R.J. (2005). Increasing walking: How important is distance to, attractiveness, and size of public open space?. Am. J. Prev. Med..

[56] Veitch J., Salmon J., Parker K., Bangay S., Deforche B., Timperio A. (2016). Adolescents’ ratings of features of parks that encourage park visitation and physical activity. Int. J. Behav. Nutr. Phys. Act..

[57] McCormack G.R., Rock M., Toohey A.M., Hignell D. (2010). Characteristics of urban parks associated with park use and physical activity: A review of qualitative research. Health Place.

[58] Beyer K.M., Kaltenbach A., Szabo A., Bogar S., Nieto F.J., Malecki K.M. (2014). Exposure to neighborhood green space and mental health: Evidence from the survey of the health of Wisconsin. Int. J. Environ. Res. Public Health.

[59] Timperio A., Giles-Corti B., Crawford D., Andrianopoulos N., Ball K., Salmon J., Hume C. (2008). Features of public open spaces and physical activity among children: Findings from the clan study. Prev. Med..

[60] Ries A.V., Gittelsohn J., Voorhees C.C., Roche K.M., Clifton K.J., Astone N.M. (2008). The environment and urban adolescents’ use of recreational facilities for physical activity: A qualitative study. Am. J. Health Promot..

